# Correlates of experimentation with smoking and current cigarette consumption among adolescents* **

**DOI:** 10.1590/S1806-37132014000600007

**Published:** 2014

**Authors:** Amanda Gimenes Bonilha, Antonio Ruffino-Netto, Mayara Piani Sicchieri, Jorge Alberto Achcar, Antonio Luiz Rodrigues-Júnior, José Baddini-Martinez

**Affiliations:** University of São Paulo, Ribeirão Preto School of Medicine, Hospital das Clínicas, Ribeirão Preto, Brazil. University of São Paulo at Ribeirão Preto School of Medicine Hospital das Clínicas, Ribeirão Preto, Brazil; University of São Paulo, Ribeirão Preto School of Medicine, Hospital das Clínicas, Ribeirão Preto, Brazil. Department of Social Medicine, University of São Paulo at Ribeirão Preto School of Medicine, Ribeirão Preto, Brazil; University of São Paulo, Ribeirão Preto School of Medicine, Ribeirão Preto, Brazil. University of São Paulo at Ribeirão Preto School of Medicine, Ribeirão Preto, Brazil; University of São Paulo, Ribeirão Preto School of Medicine, Department of Social Medicine, Ribeirão Preto, Brazil. Department of Social Medicine, University of São Paulo at Ribeirão Preto School of Medicine, Ribeirão Preto, Brazil; University of São Paulo, Ribeirão Preto School of Medicine, Department of Social Medicine, Ribeirão Preto, Brazil. Department of Social Medicine, University of São Paulo at Ribeirão Preto School of Medicine, Ribeirão Preto, Brazil; University of São Paulo, Ribeirão Preto School of Medicine, Ribeirão Preto, Brazil. University of São Paulo at Ribeirão Preto School of Medicine, Ribeirão Preto, Brazil

**Keywords:** Smoking, Tobacco use, Adolescent behavior, Stress, psychological

## Abstract

**OBJECTIVE:**

The aim of this study was to analyze social characteristics and stress as correlates of cigarette smoking in adolescence. The main intent was to identify elements that distinguish adolescents who had experimented with smoking and did not progress to regular smoking from those who became current smokers.

**METHODS:**

Students at 10 high schools in the city of Ribeirão Preto, Brazil, completed a questionnaire based on an instrument employed in a similar large-scale study. The students were classified as never-smokers or experimenters. The experimenters were subcategorized as having become current smokers or nonprogressors. Analyses were performed using adjusted logistic models.

**RESULTS:**

A total of 2,014 students (mean age, 16.2 ± 1.1 years; females, 53%) completed the questionnaire. We categorized 1,283 students (63.7%) as never-smokers, 244 (12.1%) as current smokers, and 487 (24.2%) as nonprogressors. We found that experimentation with smoking was associated with being held back a grade in school (OR = 1.80), alcohol intake (low/occasional, OR = 8.92; high/regular, OR = 2.64), illicit drug use (OR = 9.32), having a sibling or cousin who smokes (OR = 1.39), having a friend who smokes (OR = 2.08), and high levels of stress (in females only, OR = 1.32). Factors associated with an increased risk of transitioning from experimenter to current smoker were alcohol intake (low/occasional, OR = 3.28; high/regular, OR = 2.16), illicit drug use (OR = 3.61), and having a friend who smokes (OR = 7.20).

**CONCLUSIONS:**

Current smoking was associated with a profile of socioeconomic correlates different from that associated with experimentation only. Our data (showing that current smoking was associated with having a friend who smokes, alcohol intake, and illicit drug use) suggest the need for comprehensive approaches to discourage substance use during adolescence.

## Abstract (pt)

**OBJETIVO:**

Analisar características sociais e estresse como correlatos de consumo de cigarros na adolescência. O principal objetivo foi identificar elementos que distingam adolescentes que experimentaram cigarros e não progrediram para o tabagismo regular daqueles que se tornaram fumantes correntes.

**MÉTODOS:**

Estudantes de 10 escolas do ensino médio de Ribeirão Preto responderam um questionário baseado em instrumento empregado em um estudo com escala semelhante. Os estudantes foram classificados em indivíduos que nunca fumaram (NF) e experimentadores de cigarros (E). O grupo E foi subcategorizado em fumantes atuais e indivíduos que não progrediram para tabagismo corrente. As análises foram realizadas usando modelos logísticos ajustados.

**RESULTADOS:**

Um total de 2.014 estudantes (16,2 ± 1,1 anos; mulheres, 53%) responderam o questionário. Categorizamos 1.283 alunos (63,7%) em nunca fumantes, 244 (12,1%) como fumantes atuais e 487 (24,2%) como não progressores. A experimentação de cigarros foi associada a repetição de anos na escola (OR = 1,80), consumo de álcool (baixo/ocasional, OR = 8,92; intenso/frequente, OR = 2,64), uso de drogas ilícitas (OR = 9,32), fumantes entre irmãos ou primos (OR = 1,39), fumantes entre amigos (OR = 2,08) e níveis elevados de estresse (apenas em mulheres, OR = 1,32). Fatores associados com um risco maior de passar de experimentador de cigarros para fumante atual foram consumo de álcool (baixo/ocasional, OR = 3,28; regular/elevado, OR = 2,16), uso de drogas ilícitas (OR = 3,61) e amigos fumantes (OR = 7,20).

**CONCLUSÕES:**

O tabagismo atual associou-se com um perfil de correlatos socioeconômicos diferentes daqueles associados apenas à experimentação. Nossos achados (tabagismo atual associou-se a amigos fumantes, consumo de álcool e de drogas ilícitas) sugerem a necessidade de abordagens abrangentes para o desencorajamento do uso dessas substâncias na adolescência.

## Introduction

Despite the known noxious effects of cigarette smoking on health, the number of adolescent smokers has recently increased in several developing countries.^(^ 1 ^-^ 3 ^)^ In addition, it is well recognized that the majority of the smokers begin smoking when they are adolescents, and the investigation of factors related to adolescent smoking therefore assumes great importance. 

A substantial number of psychological and environmental characteristics are now recognized as being associated with smoking initiation during adolescence, including low socioeconomic status, minimal support for family bonding, smoking among peers, and peer pressure.^(^ 4 ^-^ 8 ^)^ However, most of the available studies on the smoking-related features of adolescence involve comparisons between current smokers and nonsmokers or between experimenters and non-experimenters. Little information exists on correlates of current smoking or regular smoking among adolescents, as opposed to previous experimentation only. This is particularly true concerning adolescents in Brazil, among whom there have been no studies evaluating that last aspect. 

Among several potential psychological influences, high levels of stress have been associated with smoking in adolescents.^(^ 9 ^,^ 10 ^)^ Adolescence is a period of life often characterized by adjustment problems, and the modern lifestyle can impose additional psychological burdens on youth. The 10-item Perceived Stress Scale (PSS-10) is a tool that assesses the degree to which individuals feel that situations in their lives are stressful ("unpredictable", "out of control", or "overwhelming").^(^ 11 ^)^ The PSS-10 has been employed in previous studies of adolescent smoking. In one study, the highest scores on the PSS-10 were associated with current smoking, whereas the intermediate scores were associated with experimentation only and the lowest scores were associated with being a never-smoker.^(^ 10 ^)^ Within this context, determining whether high levels of perceived stress play a role in experimentation with smoking and regular cigarette consumption in adolescence is highly relevant. 

The present study aimed to investigate levels of perceived stress and various social characteristics as correlates of cigarette smoking in high school students in Brazil. We adopted an approach that sought to identify elements that would allow a distinction to be made between adolescents who had experimented with smoking and those who had never smoked. In addition, we attempted to draw a distinction between adolescents who experimented with smoking but did not progress to regular consumption and those who became current smokers. Our hypothesis was that the profile of correlates would differ between the last two groups.

## Methods

This was a cross-sectional observational study involving adolescent students attending high school in Ribeirão Preto, Brazil, a city of approximately 600,000 inhabitants. In calculating the sample size, our objective was to attain a power of 90%, with a sample error of 5% and a confidence interval of 95%, assuming a smoking prevalence of 5.3%, as estimated on the basis of the findings of an initial pilot study. This resulted in a required sample size of 2,000 subjects. The study was approved by the Research Ethics Committee of the University of São Paulo at Ribeirão Preto School of Medicine *Hospital das Clínicas*, by the São Paulo State Board of Education, and by the principal of each of the schools involved.

Among the 56 high schools in the area under study, we randomly selected 10 (9 public schools and 1 private school). Within each of those 10 schools, we randomly selected six classes of students to be interviewed, two for each of the three grades (tenth, eleventh, and twelfth). Students were initially invited to participate in the study, and each was given a consent form to be signed at home by one of their parents or legal guardians. A few days later, participants completed a multiple choice, self-report, anonymous questionnaire, during regular class time. This instrument, developed by the authors, included items from the Fagerström Test for Nicotine Dependence (FTND)^(^ 6 ^)^ and the PSS-10,^(^ 11 ^,^ 12 ^)^ as well as items related to family social conditions, but was primarily based on the questionnaire employed in the Global Youth Tobacco Surveillance study organized by the US Centers for Disease Control and Prevention.^(^ 13 ^)^

A total of 2,044 students were invited to participate in the study. Nine students either did not produce a signed consent form or declined to participate in the survey. Of the 2,035 questionnaires distributed, 21 were returned incomplete and were therefore excluded from analysis. Consequently, the final sample comprised 2,014 completed questionnaires. 

The students were initially categorized, by smoking status, as never-smokers (those who had never smoked cigarettes) or experimenters (those who had tried cigarettes at least once, regardless of their present smoking status). The experimenters were subcategorized as current smokers or nonprogressors (those who had and had not smoked a cigarette within the last month, respectively). The definition of a current smoker was the same at that employed in the Global Youth Tobacco Surveillance study.^(^ 13 ^)^

### Measures

Based on previous reports in the literature,^(^ 4 ^-^ 8 ^)^ we selected a set of sociodemographic and socioeconomic characteristics for evaluation as dichotomous or categorical variables:

- Religion-Students were classified as religious (belonging to any organized religion or professing any type of religious conviction) or non-religious.
- Being held back in school-Students were classified as ever having been held back a grade or as never having been held back a grade.
- Personal income-Students were classified as having or not having a personal income (from a regularly paid activity or employment).
- Alcohol intake-Regarding their consumption of alcoholic beverages, students were classified as abstinent; consuming low quantities or on an occasional basis (low/occasional intake); or consuming large quantities (high intake, getting drunk sporadically) or on a regular basis (regular intake, at least once a week).
- Illicit drug use-Students were classified as users or non-users of illicit drugs (cannabis, cocaine, crack, or similar substances), those who reported even sporadic use of such substances being classified as users.
- Smoking by parents/guardians-Students were classified as having or not having at least one parent or guardian who smoked.
- Family conditions-Regarding family structure and the support available, students were classified as having two living, cohabitating parents; having two living parents who were not cohabitating; or having at least one parent who had died.
- Education of parents/guardians-For each student, the level of education of the head of their household was classified as some college (> 12 years of schooling, having earned a degree or not); some high school (9-12 years of schooling, having graduated or not); or some elementary/middle school (≤ 9 years of schooling, having completing it or not).
- Smoking by siblings/cousins-Students were classified as having or not having at least one sibling or cousin who smoked.
- Smoking by friends-Students were classified as having or not having at least one friend who smoked.
- Socioeconomic class-On the basis of self-reported data related to a number of variables, including the number and type of electrical appliances at home and the level of education of their parents, students were classified as belonging to one of the four socioeconomic classes defined by the Brazilian Association of Survey Firms (A, B, C, and D, A being the highest).
- Smoking rules at home-Students were classified as living in a home where smoking was allowed or living in a home where smoking was not allowed.
- Classes on smoking-Students were classified as having or not having attended classes or lectures dealing with the health effects of smoking.
- Level of perceived stress-On the basis of the median PSS-10 score obtained for the sample as a whole, students were classified as having a high or low level of perceived stress (individual PSS-10 scores of ≥ 17 or < 17, respectively).

### Data analysis 

Associations between characteristics and smoking status were investigated using logistic models adjusted for all features. These models allowed estimation of risk even when the outcome variable was binary. We analyzed the data using the PROC LOGISTIC function of the SAS/STAT software, version 9.0 (SAS Institute Inc., Cary, NC, USA). The level of significance adopted was p ≤ 0.05.

## Results

The clinical and sociodemographic characteristics of the adolescents are listed in Table 1. Of the 2,014 students who completed the questionnaire, 1,067 (53%) were female and 947 (47%) were male. The mean age of the sample as a whole was 16.2 ± 1.1 years. The median PSS-10 score for the sample as a whole was 17 (range, 0-40). The median of the FTND scores for the current smokers was 0 (range, 0-8). Of the 244 students classified as current smokers, 190 (77.9%) had FTND scores ≤ 2, which are indicative of very low levels of nicotine addiction.

**Table 1 -:** Sociodemographic and clinical characteristics of 2,014 high school students in the city of Ribeirão Preto, Brazil.

Characteristic	Category	n (%)
Gender	Female	1067 (53)
	Male	947 (47)
Smoking status	Never-smoker	1283 (63.7)
	Current smoker	244 (12.1)
	Nonprogressor	487 (24.2)
	Experimenter	731 (36.3)
Age, years	≤ 14	96 (4.8)
	15	476 (23.6)
	16	683 (33.9)
	17	591 (29.3)
	≥ 18	168 (8.3)
Religious	No	265 (13.2)
	Yes	1749 (86.8)
Held back a grade in school	No	1691 (84)
	Yes	323 (16)
Personal income	No	1544 (77)
	Yes	470 (23)
Alcohol intake	None	687 (34.1)
	Low or occasional	1085 (53.9)
	Heavy or regular	242 (12)
Illicit drug use	Never	1898 (94.2)
	Ever	116 (5.8)
Parent/guardian who smokes	No	1164 (58.5)
	Yes	827 (41.5)
Parental conditions	I	1315 (65.3)
	II	554 (27.5)
	III	145 (7.2)
Parent/guardian years of schooling	≤ 9	619 (35.6)
	9-12	727 (41.8)
	> 12	392 (22.6)
Sibling or cousin who smokes	No	1179 (58.5)
	Yes	835 (41.5)
Friend who smokes	No	715 (35.5)
	Yes	1299 (64.5)
Socioeconomic class	A	257 (15)
	B	1118 (65.2)
	C	333 (19.4)
	D	6 (0.4)
Smoking allowed in the home	No	1374 (68.2)
	Yes	640 (31.8)
Stress level (PSS-10 score)	High (≥ 17)	1080 (53.6)
	Low (< 17)	934 (46.4)

I: both parents were alive and living together; II: both parents were alive but lived apart; III: one or both parents were dead; and PSS-10: 10-item Perceived Stress Scale.

The odds ratios and 95% confidence intervals for the risk factors associated with experimenting with smoking are listed in Table 2. The risk of experimenting with smoking was significantly higher for the students who had been held back a grade (OR = 1.8); had low/occasional or high/regular alcohol intake (OR = 8.92 and OR = 2.64, respectively); engaged in illicit drug use (OR = 9.32); had a sibling or cousin who smoked (OR = 1.39); had a friend who smoked (OR = 2.08); or had a high level of perceived stress (OR = 1.32). 

**Table 2 -:** Risks for experimentation with smoking, by variable, among high school students in the city of Ribeirão Preto, Brazil.

Variable	Comparison	OR	95% CI	p
Gender	Male vs. Female	1.08	0.85-1.38	0.52
Age, years	15 vs. ≤ 14	0.97	0.52-1.82	0.92
	16 vs. ≤ 14	0.98	0.53-1.81	0.95
	17 vs. ≤ 14	0.98	0.53-1.81	0.94
	≥ 18 vs. ≤ 14	1.35	0.65-2.79	0.42
Religious	Yes vs. No	1.05	0.74-1.49	0.78
Held back a grade in school	Yes vs. No	1.80	1.28-2.53	< 0.01
Personal income	Yes vs. No	0.81	0.62-1.06	0.13
Alcohol intake	Low/occasional vs. None	8.92	5.83-13.64	< 0.01
	High/regular vs. None	2.64	1.99-3.51	< 0.01
Illicit drug use	Ever vs. Never	9.32	4.48-19.39	< 0.01
Parent/guardian who smokes	Yes vs. No	0.96	0.76-1.22	0.74
Parental conditions	I vs. II	1.20	0.92-1.56	0.18
	I vs. III	0.89	0.57-1.40	0.62
Parent/guardian years of schooling	> 12 vs. ≤ 9	1.10	0.77-1.57	0.59
	≤ 12 vs. ≤ 9	1.02	0.75-1.38	0.91
Sibling or cousin who smokes	Yes vs. No	1.39	1.10-1.75	< 0.01
Friend who smokes	Yes vs. No	2.08	1.60-2.71	< 0.01
Socioeconomic class	A vs. D	0.65	0.10-4.24	0.65
	B vs. D	0.63	0.10-3.99	0.62
	C vs. D	0.68	0.11-4.30	0.68
Smoking allowed in the home	Yes vs. No	1.19	0.93-1.52	0.17
Stress level (PSS-10 score)	High (≥ 17) vs. Low (< 17)	1.32	1.04-1.68	0.02

I: both parents were alive and living together; II: both parents were alive but lived apart; III: one or both parents were dead; and PSS-10: 10-item Perceived Stress Scale.

An analysis by gender showed that, among the male students, having experimented with smoking was significantly associated with being held back a grade (OR = 1.66), low/occasional and high/regular alcohol intake (OR = 10.05 and OR = 3.34, respectively), illicit drug use (OR = 11.31), and having a friend who smoked (OR = 1.96). Similar results were obtained for females, among whom having experimented with smoking was also significantly associated with having experienced the death of a parent (OR = 1.44), having a sibling or cousin who smoked (OR = 1.41), and having a high level of perceived stress (OR = 1.52).

The odds ratios and 95% confidence intervals for the risk of transitioning from an experimenter to a current smoker are listed in Table 4. The results obtained with the adjusted model show that the risk of an experimenter becoming a current smoker was significantly higher in the presence of the factors low/occasional or high/regular alcohol intake (OR = 3.28 and OR = 2.16, respectively), illicit drug use (OR = 3.61), and having a friend who smoked (OR = 7.20). 

**Table 3 -:** Risks for experimentation with smoking, by variable and by gender, among high school students in the city of Ribeirão Preto, Brazil.

Variable	Comparison	Male			Female		
		OR	95% CI	p	OR	95% CI	p
Age, years	15 vs. ≤ 14	0.92	0.36-2.38	0.86	0.90	0.38-2.14	0.81
	16 vs. ≤ 14	0.70	0.28-1.76	0.45	1.15	0.50-2.67	0.75
	17 vs. ≤ 14	0.76	0.30-1.93	0.56	1.06	0.45-2.47	0.89
	≥ 18 vs. ≤ 14	1.25	0.43-3.69	0.68	1.28	0.47-3.51	0.63
Religious	Yes vs. No	0.80	0.49-1.31	0.38	1.48	0.87-2.51	0.15
Held back a grade in school	Yes vs. No	1.66	1.03-2.67	0.04	2.11	1.28-3.46	< 0.01
Personal income	Yes vs. No	0.80	0.55-1.18	0.26	0.81	0.55-1.18	0.27
Alcohol intake	Low/occasional vs. None	10.05	5.53-18.25	< 0.01	8.40	4.50-15.70	< 0.01
	High/regular vs. None	3.34	2.19-5.12	< 0.01	2.03	1.37-2.99	< 0.01
Illicit drug use	Ever vs. Never	11.31	4.59-27.89	< 0.01	6.41	1.74-23.69	< 0.01
Parent/guardian who smokes	Yes vs. No	0.76	0.53-1.09	0.13	1.18	0.86-1.63	0.31
Parental conditions	I vs. II	0.92	0.44-1.93	0.82	0.88	0.49-1.57	0.66
	II vs. III	0.98	0.66-1.46	0.93	1.44	1.01-2.06	0.05
Parent/guardian years of schooling	> 12 vs. ≤ 9	1.37	0.78-2.40	0.28	1.05	0.66-1.68	0.84
	≤ 12 vs. ≤ 9	1.32	0.81-2.15	0.27	0.87	0.58-1.31	0.51
Sibling or cousin who smokes	Yes vs. No	1.42	0.99-2.04	0.06	1.41	1.02-1.94	0.04
Friend who smokes	Yes vs. No	1.96	1.32-2.93	< 0.01	2.32	1.62-3.32	< 0.01
Socioeconomic class	A vs. C & D	0.66	0.32-1.35	0.25	1.15	0.61-2.19	0.67
	B vs. C & D	0.66	0.40-1.10	0.11	1.18	0.78-1.76	0.44
Smoking allowed in the home	Yes vs. No	1.15	0.80-1.67	0.45	1.19	0.85-1.67	0.30
Stress level (PSS-10 score)	High (≥ 17) vs. Low (< 17)	1.17	0.82-1.66	0.38	1.52	1.08-2.13	0.02

I: both parents were alive and living together; II: both parents were alive but lived apart; III: one or both parents were dead; and PSS-10: 10-item Perceived Stress Scale..

**Table 4 -:** Risks for transitioning from experimentation to current smoking, by variable, among high school students in the city of Ribeirão Preto, Brazil

Variable	Comparison	OR	95% CI	p
Gender	Male vs. Female	1.28	0.86-1.90	0.22
Age, years	15 vs. ≤ 14	0.89	0.27-2.96	0.85
	16 vs. ≤ 14	0.90	0.28-2.89	0.86
	17 vs. ≤ 14	1.19	0.37-3.76	0.77
	≥ 18 vs. ≤ 14	1.03	0.29-3.73	0.96
Religious	Yes vs. No	1.02	0.61-1.70	0.95
Held back a grade in school	Yes vs. No	1.11	0.69-1.79	0.68
Personal income	Yes vs. No	1.17	0.77-1.78	0.45
Alcohol use	Low/occasional vs. None	3.28	1.57-6.85	< 0.01
	High/regular vs. None	2.16	1.09-4.29	0.03
Illicit drug use	Ever vs. Never	3.61	2.12-6.14	<0.01
Parent/guardian who smokes	Yes vs. No	1.22	0.82-1.81	0.32
Parental conditions	I vs. II	0.92	0.60-1.40	0.70
	I vs. III	1.91	0.95-3.83	0.07
Parent/guardian years of schooling	> 12 vs. ≤ 9	1.32	0.73-2.41	0.36
	≤ 12 vs. ≤ 9	1.35	0.81-2.26	0.25
Sibling or cousin who smokes	Yes vs. No	1.14	0.77-1.68	0.52
Friend who smokes	Yes vs. No	7.20	3.19-16.28	< 0.01
Socioeconomic class	A vs. D	0.23	0.01-4.92	0.35
	B vs. D	0.19	0.01-3.77	0.27
	C vs. D	0.27	0.01-5.42	0.39
Smoking allowed in the home	Yes vs. No	1.09	0.73-1.61	0.69
Stress level (PSS-10 score)	High (≥ 17) vs. Low (< 17)	0.84	0.56-1.25	0.38

I: both parents were alive and living together; II: both parents were alive but lived apart; III: one or both parents were dead; and PSS-10: 10-item Perceived Stress Scale.

## Discussion

The results of the present study indicate that, among adolescents in Brazil, the risk of experimenting with smoking is significantly higher for those who have been held back a grade in school, those who consume alcoholic beverages, those who use illicit drugs, those who have a sibling or cousin who smokes, those who have a friend who smokes, and those who have a high level of perceived stress. These factors have previously been recognized as being associated with such experimentation.^(^ 4 ^-^ 10 ^)^ The most relevant findings of our study were the distinctions between the experimenters who evolved to current smoking and those who did not. The risk factors found to be significantly associated with transitioning from experimentation to current smoking were alcohol intake, illicit drug use, and having a friend who smokes. These results suggest that the use of other addictive substances and peer pressure play important roles in the establishment of nicotine addiction.

The very low degree of nicotine addiction exhibited by the current smokers identified in the present study is likely attributable to the short smoking histories of the students evaluated. Nicotine acts on the central nervous system by binding to nicotinic acetylcholine receptors (nAChRs), which are ligand-gated ion channels. ^(^ 14 ^)^ Repeated exposure of nAChRs to nicotine leads to neuroadaptation to some of its effects. Simultaneous to the neuroadaptation, there is an increase in the number of nAChRs in the brain, as a response to nicotine-mediated desensitization. These mechanisms appear to play important roles in the development of nicotine tolerance and, as a consequence, in the process of increasing consumption of cigarettes over the following years.^(^ 15 ^)^

The occurrence of stressful events within the framework of the family, friendships, and school has been associated with adolescent smoking in diverse cultural settings.^(^ 9 ^,^ 10 ^)^ In the present study, we found that the risk of having a high level of perceived stress was greater among the experimenters than among the never-smokers, a finding that is in agreement with those of previous studies.^(^ 9 ^)^ It can therefore be assumed that stressors and a high level of perceived stress are major determinants of whether or not an adolescent will experiment with smoking. Our results also indicate that certain stressors are stronger determinants of such experimentation in females than in males. In fact, there are data in the literature indicating that stressful events in life may be more important contributors to smoking initiation in females than in males. ^(^ 16 ^,^ 17 ^)^ In the present study, the proportion of females who had at least one dead parent was higher among the experimenters than among the never-smokers, which might have contributed to our finding that PSS-10 scores were higher among the female experimenters than among their male counterparts. Among adult smokers, it has been shown that women are more likely than are males to report tension reduction as a reason for smoking.^(^ 18 ^)^ The available evidence, therefore, implies that emotional phenomena play a more important role in experimentation with smoking in females than in males.

In our sample, the PSS-10 scores were higher among the experimenters than among the never-smokers. However, that measure did not allow us to make a clear distinction between the experimenters who became nonprogressors and those who became current smokers. The finding suggests that high levels of perceived stress do not fully explain the progression to nicotine addiction. This is also evidence that continuous smoking itself is not associated with progressive increases of perceived stress in adolescence. 

Various studies have demonstrated strong associations between smoking and alcohol abuse in adolescents and adults.^(^ 19 ^,^ 20 ^)^ This is also true for smoking and illicit drug use.^(^ 21 ^)^ In fact, the presence of smoking in adolescents is recognized as a predictor of alcoholism and illicit drug use in young adults.^(^ 22 ^)^ Although the true nature of these associations is not yet fully understood, genetic and environmental factors appear to contribute to individual outcomes in a complementary manner. ^(^ 23 ^)^ In addition, psychoactive drugs might promote their effects through the same neural systems.^(^ 23 ^)^

Some features of adolescent behavior, such as risk taking and sensation seeking, have been traditionally considered vulnerability factors for the development of substance abuse disorders. The human brain could be hard-wired for sensation seeking and risk taking during adolescence.^(^ 24 ^)^ As the adolescents become older, they would develop the skills to better regulate their emotions and impulsiveness. Repeated exposure of a still-developing brain to nicotine could certainly lead to structural changes, including the excessive expression of nAChRs and impairment of the ability to regulate impulsiveness. Recent studies have suggested that nAChRs play an important role not only in nicotine addiction but also in dependence on alcohol and other drugs.^(^ 25 ^)^ Therefore, we can hypothesize that adolescent smoking constitutes a major contributor to the establishment of other addictions in youth. Regardless of the mechanisms involved, the findings of the present study confirm the need to adopt more comprehensive approaches to designing prophylactic and therapeutic interventions to prevent the use of all drugs during adolescence. 

We found that, in comparison with the never-smokers, the experimenters were more likely to have a friend who smoked, as well as to have a sibling or cousin who smoked. The latter association was strong among the females. However, it is likely to be valid for males as well, despite the fact that, among the males evaluated here, the association between experimenting with smoking and having a sibling or cousin who smoked reached only borderline significance (p = 0.06). Although having a friend who smoked was identified as a risk factor for transitioning from experimenter to current smoker, a history of smoking among close relatives, including parents/guardians and siblings/cousins, was not. This emphasizes the great potential importance of peer pressure, coupled with the need for acceptance into and identification with groups of all kinds, for the maintenance of the addiction. Continuous interaction with friends who smoke not only can serve as a positive stimulus to keep adolescents smoking but also could inhibit impulses and initiatives for quitting, at their inception. Our findings suggest the need to develop new forms of intervention that target groups of friends, rather than individuals. New forms of media communication through virtual networks, which are so popular among adolescents, could be an appropriate medium for such innovative interventions. 

The present study has substantial limitations, including its cross-sectional design and the fact that it relies solely on self-reported data. However, recent studies strongly suggest that young people provide reliable information about their consumption of cigarettes and drugs.^(^ 26 ^)^ In addition, some of the students who were classified as current smokers might in fact be nonprogressors in transition. Furthermore, some of the students classified as nonprogressors might have been more accurately classified as former smokers, depending on the quantity of cigarettes previously smoked. Nevertheless, because the mean age of the sample was low, any former smoker erroneously classified as a nonprogressor would have a short smoking history and the misclassification would therefore be unlikely to change the final results in any substantial way. Finally, we did not have access to adolescents who were not regular students in the school system. Despite these limitations, our results contribute to deepening the knowledge of risk factors for smoking among adolescents in Brazil, a topic on which there have been only a few robust studies.^(^ 27 ^-^ 29 ^)^

In conclusion, although high levels of perceived stress and various social elements are clearly associated with experimentation with smoking, only having friends who smoke, consuming alcoholic beverages, and using illicit drugs appear to be important markers for identifying experimenters who are at a high risk of becoming current smokers. The results of the present study suggest the need to adopt more comprehensive approaches to health interventions dealing with the use of multiple substances during adolescence.
